# Seropositivity for West Nile Virus Antibodies in Patients Affected by Myasthenia Gravis

**DOI:** 10.14740/jocmr2413w

**Published:** 2016-01-26

**Authors:** Marilena Greco, Pietro Cofano, Giambattista Lobreglio

**Affiliations:** a Clinical Pathology Laboratory, “Vito Fazzi” Hospital, ASL Lecce, Lecce, Italy

**Keywords:** Myasthenia gravis, Autoimmunity, West Nile virus

## Abstract

**Background:**

Myasthenia gravis (MG) is an autoimmune neuromuscular disease characterized by varying degrees of weakness of the skeletal muscles. Specific auto-antibodies against acetylcholine receptor (AChR) are present in the majority of MG patients, although the main cause behind its development still remains unclear. Recently MG development following West Nile virus (WNV) infection has been described in patients without any earlier evidence of MG. It is known that infectious agents trigger immune response and occasionally initiate autoimmune disease. WNV, the causative agent of both benign illness and neuroinvasive disease, has become endemic in many countries in all continents.

**Methods:**

In the present study, 29 patients (15 males and 14 females, 19 - 78 years old) with confirmed diagnosis of MG and elevated levels of AChR autoantibodies were screened for the presence of serum anti-WNV antibodies and compared to a similar population affected by different autoimmune diseases. Indirect immunofluorescent antibody technique was used to evaluate the reaction of patients’ sera on cells infected by WNV.

**Results:**

Positive fluorescent signals for anti-WNV IgG were obtained in 17% of MG patients, although no clinical manifestations related to WNV infection were reported. These results are in agreement with previous data and appear of great interest in the understanding of the pathogenic autoimmune mechanisms at the bases of MG development.

**Conclusion:**

As already observed in other human autoimmune diseases, pathogen-triggered autoimmunity could be involved in MG by breaking immunological self-tolerance through possible mechanisms of molecular mimicry between virus proteins and AChR subunits. In predisposed individuals, WNV infection could also represent an additional risk factor to initiate MG.

## Introduction

Myasthenia gravis (MG) is a chronic autoimmune disease affecting neuromuscular transmission characterized by painless weakness of skeletal muscles, which develops or becomes more pronounced upon physical exertion. MG may affect any voluntary muscle, although muscles that control eye and eyelid movements, facial expression, and swallowing are most frequently affected. The onset of the disorder may be sudden and symptoms often are not immediately recognized as MG. Precise basis of MG development was not demonstrated so far; interestingly recently its development has been associated to virus infection such as West Nile virus (WNV) infection [1]. MG occurs in all ethnic groups and both genders; annual incidence of MG has been estimated 30/1,000,000. Overall, incidence rates have increased over time owing to increasing of average age of patients, a greater awareness of the disease and improved methods of diagnosis [2]. MG can occur at any age, and most commonly affects young adult women with a disease peak in the third decade, while in men it has a bimodal distribution, with peak in third and sixth decades [3, 4]. MG occurs in 40% of patients affected by thymoma, in this case with peaks in fourth and fifth decades and female predominance [5]. The clinical severity is graded from ocular signs only, to generalized myasthenia (mild or moderate), to severe generalized disease and myasthenic crisis [6, 7].

MG is caused by a defect in the transmission of nerve impulses to muscles. Normal communication between the nerve and muscle at the neuromuscular junction is primarily impaired by autoantibodies against acetylcholine receptors (anti-AChRs). Activated CD4^+^ T-helper cells drive the autoimmune response in MG. Several mechanisms are involved in neuromuscular transmission impairment including functional blockade of AChR, increased degradation of AChR, and complement-mediated destruction of post-synaptic folds [8]. Autoantibodies against AChR are detected in 90% of patients with generalized MG, and about 50% of ocular disease [9], and their presence is not correlated with the severity of disease. When anti-AChRs are undetectable, MG is termed seronegative. Autoantibodies against muscle-specific kinase (MuSK), a protein that helps organize AChRs on the muscle cell surface, are specific for seronegative MG and correlate to the severity of disease [10-12]. Other antibodies detected in MG are striational antibodies, which target sarcomeric protein of striated muscle (titin, ryanodine receptor, myosin, and actin) [13], and it is known that these antibodies are associated with the presence of thymoma.

The exact cause of MG is not known yet. One hypothesis is that the condition may be triggered by a virus or other infection that has a similar structure to a part of the AChR. The antibodies that the immune system produces to fight the virus afterward also mistakenly attack the receptors. Recent findings described the development of MG following WNV infection in a group of patients without any earlier evidence of MG [1].

WNV has been previously reported in patients with various neuromuscular diseases that presumably involve autoimmune mechanisms [14-18]. WNV, a mosquito-borne RNA flavivirus and human neuropathogen, is the causative agent of benign West Nile fever (75% of infected people have asymptomatic infection and 25% have influenza-like symptoms) or West Nile neuroinvasive disease in humans (< 1% of infected people); it is responsible for epidemic viral encephalitis and an important cause of neurological disease. WNV is transmitted mainly through the bite of infected mosquito; birds are identified as a major host, horses are commonly infected and human beings are dead-end host. Human-to-human transmission is rare; however, it can occur through organ transplantation, blood transfusion, breastfeeding or percutaneous inoculation. WNV was first isolated in 1937 from a febrile woman in the West Nile region of Uganda; thereafter, the virus became recognized as a cause of human meningitis or encephalitis. Large human epidemics occurred in different geographical areas such as Africa, the Middle East, Europe, Russia, and North America. In 2011, human cases were reported in Albania, Greece, Israel, Italy, Romania, Russia, and Mexico. At present, the geographic range of WNV includes Africa, Asia, Europe, Australia, North America, and South America [19]. WNV has become endemic in many countries in all continents and the diagnosis of WNV infection should be considered in any patient with an unexplained acute febrile or neurological illness during the summer months, particularly if recently exposed to mosquitoes. WNV infection might be transmitted through blood transfusion and solid organ transplantation.

Besides acute morbidity and mortality, long-term sequelae of WNV-related illness (such as physical, cognitive or psychological, and functional sequelae) have been associated to virus infection as recently reviewed by Patel et al [20].

It is widely known that viral infections have been often associated to autoimmune diseases [21]. Autoimmunity ranks as the third most prevalent cause of morbidity and mortality in the Western World. Genome wide association studies have identified polymorphisms in numerous genes associated with immune activation and regulation predisposing to the development of autoimmune disease [22, 23]; however, manifestation of autoimmunity involves a combination of other factors including environmental ones and virus infection [21]. In fact, viruses may trigger immune response, breakdown self-tolerance and determine immune-mediated attack directed against both viral and self-antigens. Traditionally, cross-reactive B- and T-cell recognitions, known as molecular mimicry, as well as bystander T-cell activation, culminating in epitope spreading, have been the main mechanisms elucidated through which infection may culminate in T- and B-cell-mediated autoimmune response.

## Methods

In the present study, 29 patients (15 males and 14 females of age from 19 to 78 years old) from the South of Apulia (Italy), with confirmed diagnosis of MG and elevated AChR autoantibodies were screened for the presence of serum anti-WNV antibodies.

A further subset of 20 patients affected by different autoimmune diseases with seropositivity for different autoantibodies than AChR were tested for anti-WNV antibodies (12 patients seropositive for anti-nuclear antibodies with different nuclear patterns, four for anti-parietal cells antibodies, two for antibodies against liver/kidney/microsomal antigens, and two for anti-smooth muscle antibodies).

Indirect immunofluorescent antibody technique was used to evaluate the reaction of patients’ sera on cells infected by WNV, according to manufacturer directions (anti-West Nile virus indirect immunofluorescent antibody (IFA), Euroimmun, Lubeck, Deutschland). Shortly, 30 μL of 1:10 diluted patients sera were incubated on the substrate (WNV-infected and non-infected cells for each sample) for 30 min, than washed twice with PBS-Tween for 5 min, incubated with 25 μL of fluorescein-conjugated secondary antibody (anti-human IgG) for 30 min and finally washed with PBS-Tween for 5 min. Fluorescein isothiocyanate (FITC) fluorescence was observed by microscopy.

A specific reaction was excluded by contemporary testing of each patient’s sera on cells not infected by WNV (negative control).

Similar indirect immunofluorescent antibody procedures were used for detection of other autoantibodies in non-MG patients.

AChR autoantibodies were detected by quantitative radioimmunoassay according to manufacturer directions (ACHR-AB, Cisbio Bioassays, France).

## Results

**Figure 1:**
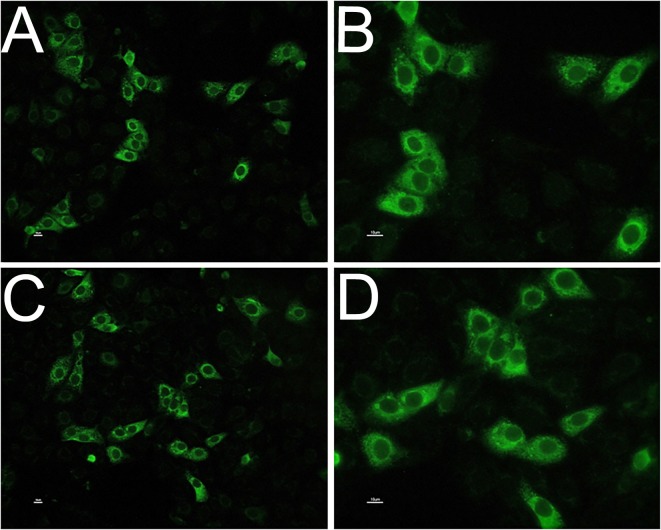
Anti-WNV antibodies in patients’ sera detected by indirect immunofluorescent antibody technique; positive immunofluorescent signal on cytoplasm of WNV-infected cells substrate, observed at × 20 (A and C) and × 40 magnification (B and D). Negative control did not show any fluorescent signals.

The presence of anti-WNV antibodies was observed in 17% of the MG patients analyzed in the current study: five of the 29 MG individuals showed a positive immunofluorescent signal on cytoplasm of WNV-infected cells substrate when their serum was incubated on the substrate (Fig. 1), thus indicating seropositivity for WNV antibodies in patients with MG in an interesting percentage of analyzed patients. Different levels of positivity were observed without correlation with the severity of the disease. No immunofluorescent signal was detected on non-infected cells for none of the analyzed sera (negative control).

Patients affected by other autoimmune diseases than MG that were tested for anti-WNV did not show fluorescent signal in the cytoplasm of the substrate cells, and nuclear staining was observed for patients seropositive for anti-nuclear antibodies.

Among positive MG patients, only one-fifth was represented by males, while in the whole group of MG patients, the number of males and females was similar (Table 1).

**Table 1:** West Nile Virus Antibodies in Patients Affected by Myasthenia Gravis

WNV antibodies in MG patients		%	Gender (M/F)	Age (years)	Immunofluorescent signal intensity
Total	N = 29	100	15/14	19 - 78	
Negative	N = 24	83	14/10	19 - 78	-
Positive	N = 5	17	1/4	60 - 77	
			F	60	+
			F	62	++
			F	77	+++
			F	65	+++
			M	62	++

None of analyzed patients showed any clinical manifestation of WNV infection. Moreover, none of them referred any travel out from the South of Italy area, besides one who referred of a travel to Greece several years before MG manifestation. The same patient was double screened for WNV antibodies at the time of our study during treatment for MG, while he did not have any symptoms, and he was still seropositive for both WNV antibodies and AChR antibodies.

## Discussion

The present study evidenced seropositivity for WNV antibodies in 17% of patients with MG, and all of them with elevated AChR autoantibodies, although none of them apparently came in contact with the virus or with the area in which the virus is more diffused. Our findings appear in accordance with recent data describing cases of MG developed after several months of WNV infection [1]. In our study, we also investigated the possible seropositivity for WNV in other autoimmune diseases than MG (such as systemic lupus erythematosus, rheumatoid arthritis, autoimmune hepatitis and atrophic gastritis), without discovering any positive patients. On the basis of these results, one can hypothesize a pathogenetic mechanism involved in the insurgence of MG based on WNV triggering of the patient’s immune system. No evidence that a neurotropic virus such as WNV causes morphological damage to AChR subunits or the neuromuscular junction has been so far proposed. Rather, the defect in neuromuscular transmission appears to be mediated indirectly by host factors induced by virus. Several data already suggested that autoimmunity may result from numerous immune pathway triggered by several microorganisms [24]. Although a number of viruses and bacteria have been linked to the initiation of certain autoimmune diseases, identifying a particular virus or bacteria that is solely responsible for the induction of an autoimmune response is rare.

There are a variety of mechanisms proposed as pathway through which infection may culminate in B- and T-cell-mediated autoimmune response, such as molecular mimicry, bystander T-cell activation, i.e. the activation of T cells through a mechanism that is independent of specific TCR stimulation (leading to enhanced processing and presentation of self-antigens, which induces the expansion or spreading of immune response toward different self-antigens), exposure of cryptic antigens (which, following tissue injury, cell death, oxidative stress, become recognized as non-self and induce an autoimmune response) and superantigens (produced by a variety of microorganisms or virus-infected cells that can bind TCR irrespective of its antigenic specificity, resulting in the activation of a large number of T lymphocytes of different antigenic specificity, thus behaving as a potent immune-stimulating molecule) and polyclonal activation [25, 26].

Inflammation induced by exposure to a foreign antigen can lead to autoimmune diseases from cross-reactive epitopes (molecular mimicry). These epitopes are segments of foreign antigens which, when presented to either T or B cells in the context of the major histocompatibility complex (MHC), can activate CD4^+^ or CD8^+^ T cells. The induction of the immune response and subsequent proinflammatory cytokines release is critical for clearance of a virus or bacteria. However, a continued proinflammatory response against specific host tissues can occur when there is sequence or structural homology between foreign antigens and self-antigens, process known as molecular mimicry [27]. Although this idea has been associated with autoimmunity, mimicry (cross-reactivity) also provides protection for the host (heterologous immunity) [28]. Cross-reactivity or mimicry between various strains of viruses or bacteria could help to explain how protective immunity arises in certain individuals even in the absence of prior exposure to an emerging pathogen. This example of sequence homology in which molecular mimicry between viruses leads to protective immunity is in contrast to a pathogen mimicking host epitopes [29].

Linear sequence matches in amino acid motifs are not the only criteria for mimicry. It has been hypothesized that self-reactive immune cells are primed by molecular mimicry and bystander activation, thereby sensitizing the immune cells without any apparent disease. Subsequent environmental insults could induce these sensitized autoreactive cells to cause an autoimmune disease [30].

The recent observation of MG insurgence after WNV infection [1] indicates the possible involvement of this neurotropic flavirus as triggers in the autoimmune disease.

Other than WNV, two other flaviviruses have been implicated in human autoimmune diseases: hepatitis C virus [31] and Dengue virus (DENV) [25, 32]. Hepatitis C virus is associated with many extrahepatic disease manifestations, autoimmune phenomena, and frank autoimmune diseases in almost half of chronic carriers. It has been reported frequently in association with Sjogren syndrome, rheumatoid arthritis, systemic lupus erythematosus, polyarteritis nodosa, antiphospholipid syndrome, inflammatory myopathies, and sarcoidosis [31, 33]. DENV has been recently found to show molecular mimicry with coagulation factors able to induce the production of autoantibodies with biological effects similar to those due to anti-thrombin antibodies, which inhibit thrombin activity and enhance fibrinolysis, and may lead to the Dengue hemorrhagic fever and shock syndrome [34].

Recently Getts et al (2013) reviewed mechanisms by which virus infection and antiviral immunity contribute to the development of central nervous system (CNS) autoimmune disorders; certain CNS infections have been strongly correlated with autoimmune disease in humans and a strong support for virus-triggered CNS autoimmunity is derived from animal models studies like in the case of infection with flavivirus as Japanese encephalitis virus (JEV), Semliki forest virus (SFV), and Sindbis virus (SV) [21].

Interestingly, besides acute morbidity and mortality, WNV-related illness is associated to severe long-term physical, cognitive or psychological, and functional sequelae as reported by Patel et al (2015) in their systematic scientific literature review of studies on patients infected with WNV with more than 10 years of follow-up data. The most common physical sequelae were muscle weakness, fatigue and myalgia with an average percentage of about 15-20% of patients (including both those with neuroinvasive disease or those with West Nile fever). The most common cognitive sequelae were memory loss, depression, and difficulty concentrating with an average percentage of about 12-18% of patients (including both those with neuroinvasive disease or those with West Nile fever). On the other hand, absence of comorbid disorder was associated with faster recovery of both physical and mental performances. Older age (more than 50 or 65 years at infection, depending on different studies) has been associated to a higher risk of sequelae, while male gender has been reported to be more affected by WNV neuroinvasive disease. Those percentages of sequelae described above as well as average age older than 60 years appear very similar to the percentage of MG patients seropositive for WNV antibodies (17%, with an age range of 60 - 77 years) observed in the present study (Table 1). This finding suggests the possible involvement of autoimmune disease as a mechanism of lacking overall recovery from WNV-related illness and long-term sequelae. In our patients seropositivity for WNV was observed prevalently in women affected by MG rather than men (Table 1), differently from what previously reviewed by Patel et al. However, it has to be considered that our sample was restricted to patients affected by MG and it is well known that autoimmune diseases have an increased incidence in women than men [35]. Moreover all patients analyzed in our study, who developed MG, did not have any WNV neuroinvasive infection, being apparently asymptomatic for the WNV infection.

The prevalence of MG among all neuroinvasive WNV cases has been previously estimated around 1.2% [1], which is 100-fold higher than the estimated prevalence of MG in the general US population, supporting the proposed link between WNV infection and development of MG. As further confirm of this hypothesis we found 17% seropositivity for WNV antibodies in MG patients analyzed in this study. Although none of these patients showed any evidence of WNV infection, the specific association with MG can be proposed since other autoimmune disease patients did not evidence anti-WNV antibodies.

It seems plausible that WNV infection triggers the autoimmune mechanisms for MG development by pathogen-triggered breaking of immunological self-tolerance through possible mechanisms of molecular mimicry between virus proteins and AChR subunits together with other risk factors like environmental factors or genetic predisposition, as can be argued from the family history of MG patients.

At present, further studies appear to be needed to better clarify and confirm the mechanism by which the autoimmune disease evolves after silent or fully developed WNV infection.

## Abbreviations

MG: myasthenia gravis
WNV: West Nile virus
AChR: acetylcholine receptor
IFA: indirect immunofluorescent antibody
DENV: Dengue virus
CNS: central nervous system
TCR: T-cell receptor
MHC: major histocompatibility complex
FITC: fluorescein isothiocyanate
